# A Case of Proximal Humerus Fracture Dislocation Presenting With Failed Primary Open Reduction and Internal Fixation Followed by Salvage Reverse Total Shoulder Arthroplasty

**DOI:** 10.7759/cureus.29407

**Published:** 2022-09-21

**Authors:** Mina Entessari, Howard Bar-Eli, Julian Bernal

**Affiliations:** 1 Medicine, Herbert Wertheim College of Medicine, Florida International University, Miami, USA; 2 Trauma and Orthopedics, Miami Orthopedics and Sports Medicine Institute, Baptist Health South Florida, Miami, USA

**Keywords:** salvage reverse total shoulder arthroplasty, avascular necrosis humeral head, humeral head collapse, open reduction internal fixation, proximal humerus fracture-dislocation

## Abstract

The treatment of proximal humerus fractures is complex. Factors like fracture pattern, patient age, pre-injury activity level, soft tissue status, and comorbidities play a role in decision-making for non-operative versus operative management. Complications of non-operative vs operative management of proximal humerus fractures include but are not limited to arthrofibrosis, fracture nonunion, and avascular necrosis.

We report an unusual case of a 64-year-old female presenting with a three-part proximal humerus fracture dislocation. The patient underwent primary open reduction internal fixation. Twenty-four weeks after open reduction and internal fixation, the patient experienced collapse of the humeral head with intraarticular hardware migration, and she underwent hardware removal. Fifty-two weeks after hardware removal, the patient experienced avascular necrosis of the humeral head, and she underwent salvage reverse total shoulder arthroplasty. There is debate in the current literature on the best management of multi-part proximal humerus fracture-dislocations.

## Introduction

Proximal humerus fractures (PHF) are common, with a worldwide prevalence ranging from 4% to 10% [1,2]. Notably, PHF is the third most common osteoporotic fracture in populations older than 65 years old [3]. Approximately 85% of PHF occur in populations older than 50 years old [4]. PHF can be categorized using Neer’s classification, which categorizes PHF into four segments: the humeral head, the humeral shaft, the lesser tuberosity, and the greater tuberosity. The fragments can be further classified as involving the articular surface, concomitant dislocation, or displacement defined as a fracture fragment being angulated by more than 45°, separated by more than 1 cm or 5 mm for the greater tuberosity fragment [5].

There are different treatment modalities for PHF. Non-operative management includes immobilization, analgesia, and rehabilitative therapy. Operative management includes open reduction and internal fixation (ORIF), hemiarthroplasty, or reverse total shoulder arthroplasty (rTSA) [6,7].

There is debate about the best treatment option for PHF. Common factors taken into consideration when deciding the management of PHF include fracture classification, patient age, soft tissue status, pre-injury functional status, and bone quality. Minimally displaced PHF is defined as having fracture fragments with less than one centimeter of displacement or less than five millimeters of displacement of the greater tuberosity fragment. Minimally displaced PHF treated non-operatively have demonstrated good functional outcomes [7]. Multi-fragment PHF that have fracture fragments displaced greater than 1 cm, dislocated fractures, and the presence of neurovascular injuries are typically absolute indications for operative management [7]. Age also plays a large role in guiding treatment. Elderly populations defined as age ≥ 65, with three-part or four-part PHF, are recommended to undergo rTSA. On the other hand, populations aged <65, with three-part or four-part PHF, are typically recommended to undergo ORIF, if possible, to maximize shoulder function and to delay arthroplasty [8]. Rotator cuff status plays a role in operative planning. Deficient rotator cuff status often warrants rTSA [9]. Bone quality plays a factor when deciding if ORIF will be a viable treatment option. This is because lack of bone quality can lead to lack of screw fixation, which can result in possible varus collapse, screw cutout, or need for revision surgery [10]. It is integral to consider all these factors when determining the course of treatment that will result in the best patient outcomes.

Common complications of non-operative management of PHF include fracture non-union, malunion, avascular necrosis, arthrofibrosis, and post-traumatic arthrosis. Common complications of operative management of PHF include infection, nerve injury, hardware malfunction, nonunion, avascular necrosis, loss of reduction, arthrofibrosis, and post-traumatic arthrosis [6,11]. There is a lack of PHF presenting with both failure of primary ORIF and hardware removal reported in the literature, as we describe in this patient presentation.

## Case presentation

Injury presentation

**Figure 1:**
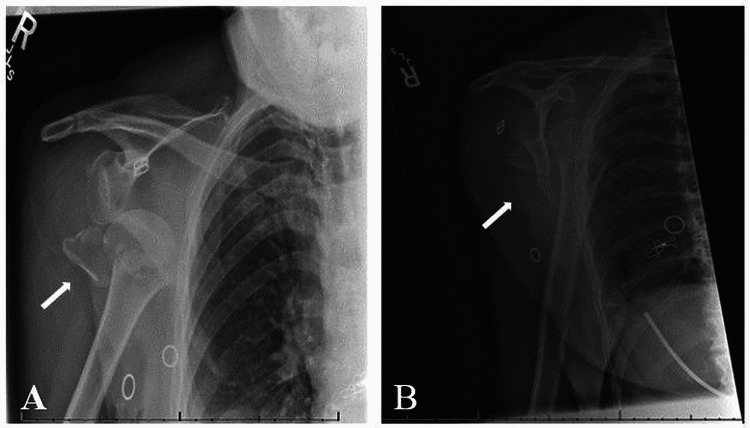
(A) Anteroposterior and (B) scapular Y X-ray views demonstrating anterior-inferior dislocation of the humeral head with impacted fracture of the humeral neck and avulsion of the greater tuberosity.

A 64-year-old right-hand dominant female presented with right shoulder pain after landing on her shoulder from a ground-level fall. There was no history of any previous injury to the right shoulder. Physical exam of the right upper extremity was remarkable for deformity of the right shoulder. X-rays of the right shoulder demonstrated anterior-inferior dislocation and three-part PHF (Figure 1). Computed tomography (CT) scan of the right shoulder was remarkable for anterior-inferior right shoulder dislocation and right surgical neck humerus fracture with impaction. The fracture extended to the greater tuberosity and had comminution. The greater tuberosity fragment measured 3.1 cm and was displaced 1.7 cm laterally. Closed reduction in the emergency department failed to achieve acceptable alignment indicating surgical fixation. The primary goal was to perform an ORIF of the right proximal humerus given the patient’s young age and high pre-injury activity level. The possibility of rTSA intraoperatively would be indicated if the patient had deficient rotator cuff status and if the degree of bone comminution did not permit proper screw fixation to achieve fracture stability and anatomic reduction.

ORIF with biceps tenodesis

The patient underwent ORIF of the right proximal humerus on the same day of her injury. Intraoperatively, the long head of the biceps was found to be entrapped within the fracture site over a piece of comminution and a biceps tenodesis was performed. The rotator cuff was found to be intact. A fibular strut allograft was utilized to achieve humeral height due to a large defect of the humeral head because of comminution. A three-hole right lateral proximal humeral locking plate was used.

**Figure 2:**
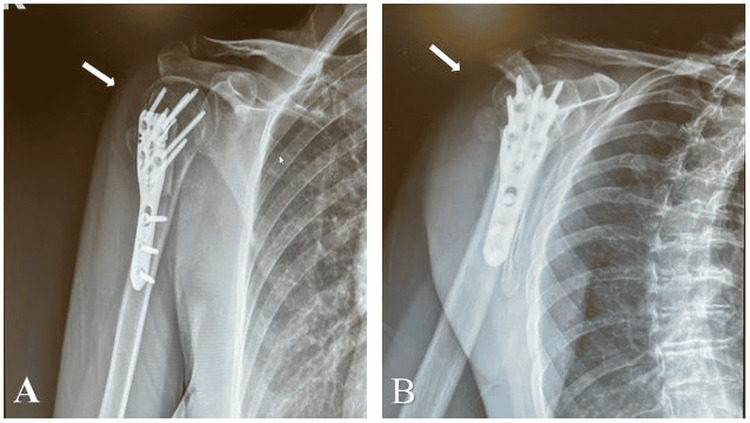
Two weeks post-ORIF (A) anteroposterior and (B) scapular Y X-ray views of right proximal humerus fracture with maintenance of fracture alignment and intact hardware. There is no intra-articular penetration of the hardware into the right glenohumeral joint. ORIF: open reduction and internal fixation

**Figure 3:**
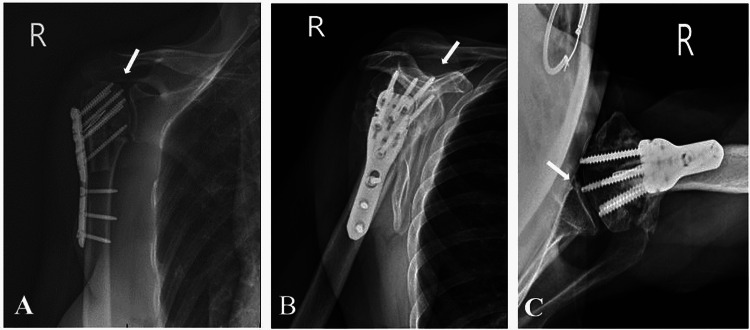
Twenty-four weeks post-ORIF (A) anterioposterior, (B) scapular Y, and (C) axillary X-ray views demonstrating maintained alignment at the fracture site. There is collapse of the humeral head articular surface with penetration of the screw tips into the glenohumeral joint. The fibular strut is visualized within the humeral canal. ORIF: open reduction and internal fixation

Two weeks postoperatively, X-rays of the right shoulder demonstrated proper fracture alignment, and intact hardware, with no intraarticular penetration of the hardware into the glenohumeral joint (Figure 2). At 24 weeks postoperatively, the patient presented with progressive anterolateral right shoulder pain and poor range of motion (Table 1). X-rays of the right shoulder at 24 weeks postoperatively demonstrated collapse of the right humeral head surface with penetration of the screws into the glenohumeral joint (Figure 3). The penetration of the screws was likely the source of the patient’s pain and poor range of motion. Given the patient was young and active, removal of the intraarticular screws or shoulder arthroplasty could allow her better function. Though the patient had no signs and symptoms of infection, erythrocyte sedimentation rate (ESR) and C-reactive protein (CRP) tests were ordered and returned normal. It was decided to proceed with the removal of hardware with manipulation under anesthesia. At that time, the patient did not desire more invasive surgery, and given her young age,, arthroplasty was still not ideal.

**Table 1:** Measured ranges of motion of the right shoulder for three different surgeries across two years. NM: not measured; AROM: active range of motion; PROM: passive range of motion; Post-op: postoperatively; T7: thoracic spine 7; T8: thoracic spine 8; L4: lumbar spine 4; L5: lumbar spine 5; S1: sacral spine 1; PSIS: posterior superior iliac spine

	Surgery 1, 2 weeks Post-op	Surgery 1, 11 weeks Post-op	Surgery 1, 24 weeks Post-op	Surgery 2, 3 weeks Post-op	Surgery 2, 8 weeks Post-op	Surgery 2, 12 weeks Post-op	Surgery 2, 20 weeks Post-op	Surgery 2, 24 weeks Post-op	Surgery 2, 52 weeks Post-op	Surgery 2, 64 weeks Post-op	Surgery 3, 2 weeks Post-op	Surgery 3, 6 weeks Post-op	Surgery 3, 12 weeks Post-op
AROM Forward Flexion	NM	60°	60°	70°	100°	75°	90°	80°	60°	40°	NM	60°	50°
AROM Abduction	NM	30°	30°	60°	45°	45°	80°	60°	60°	30°	NM	40°	90°
AROM External Rotation	NM	20°	20°	50°	50°	20°	50°	50°	50°	30°	NM	50°	50°
AROM Internal Rotation	NM	PSIS	Right Buttocks	Right Buttocks	Right Buttocks	Right Buttocks	Right Buttocks	Right buttocks	Thoracic Spine 7	Right Buttocks	NM	PSIS	Right buttocks
PROM Forward Flexion	60°	90°	60°	90°	120°	75°	110°	95°	60°	60°	90°	80°	90°
PROM Abduction	60°	45°	30°	70°	45°	60°	100°	70°	45°	70°	60°	55°	90°
PROM External Rotation	30°	20°	20°	50°	50°	20°	50°	50°	50°	30°	NM	50°	50°
PROM Internal Rotation	L5-S1	L5-S1	PSIS	Right Buttocks	Right Buttocks	Right Buttocks	L4-L5	Right Buttocks	T7-T8	Right buttocks	NM	PSIS	Right lumbar region

Hardware removal and manipulation under anesthesia

The patient underwent hardware removal of the intraarticular screws as well as manipulation under anesthesia at 29 weeks from primary ORIF. Intraoperatively, the screws concerning for intraarticular penetration were identified radiographically and removed. Additional arthrofibrosis was debrided, and the rotator cuff was found to be intact. The right shoulder was then manipulated to 120° of forward flexion, 100° of abduction, 45° external rotation, and internal rotation to her abdomen.

**Figure 4:**
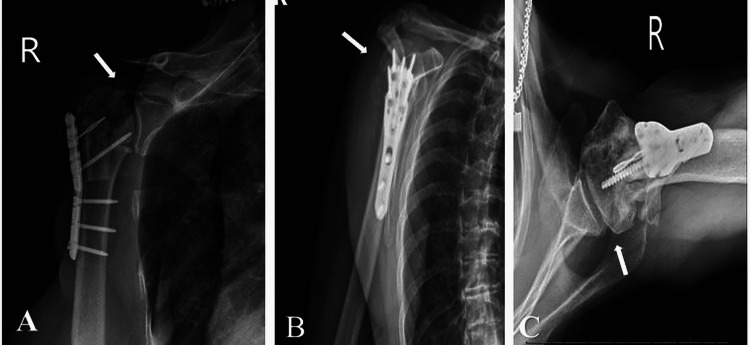
Three weeks post hardware removal and manipulation under anesthesia: (A) anteroposterior, (B) scapular Y, and (C) axillary X-ray views demonstrating collapse of the articular surface of the humeral head without change in proximal humerus alignment since removal of hardware.

**Figure 5:**
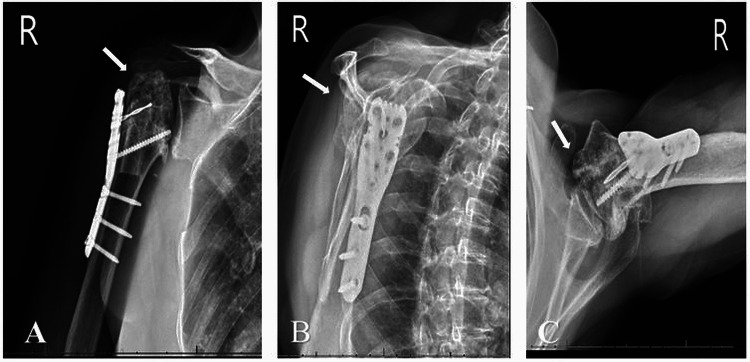
Fifty-two weeks post hardware removal and manipulation under anesthesia: (A) anteroposterior, (B) scapular Y, and (C) axillary X-ray views demonstrating progressive deformity and collapse of the humeral head with fragmentation. There is a prominent screw is within the glenohumeral joint, mild glenoid articular wear.

Three weeks postoperatively from hardware removal and manipulation under anesthesia, X-rays were remarkable for no change in humeral head alignment (Figure 4). At 20 weeks postoperatively the patient presented with worsening right shoulder pain and markedly reduced range of motion. The option of steroid injection versus total shoulder arthroplasty was discussed with the patient. The patient opted to receive a right shoulder intra-articular corticosteroid injection. Four weeks after the corticosteroid injection the patient presented with mild improvement in her right shoulder pain but had a worse range of motion (Table 1). The patient was lost to follow-up until she presented at 52 weeks postoperatively. X-rays of the right shoulder were remarkable for the collapse of the humeral head and prominent screws within the glenohumeral joint due to avascular necrosis (AVN) (Figure 5). It was discussed with the patient that due to AVN of the right shoulder, rTSA was the best option to improve her pain and range of motion. The patient opted to receive a second corticosteroid shoulder injection to provide some relief from her symptoms and to allow her more time to plan for surgery.

Hardware removal and salvage reverse total shoulder arthroplasty

**Figure 6:**
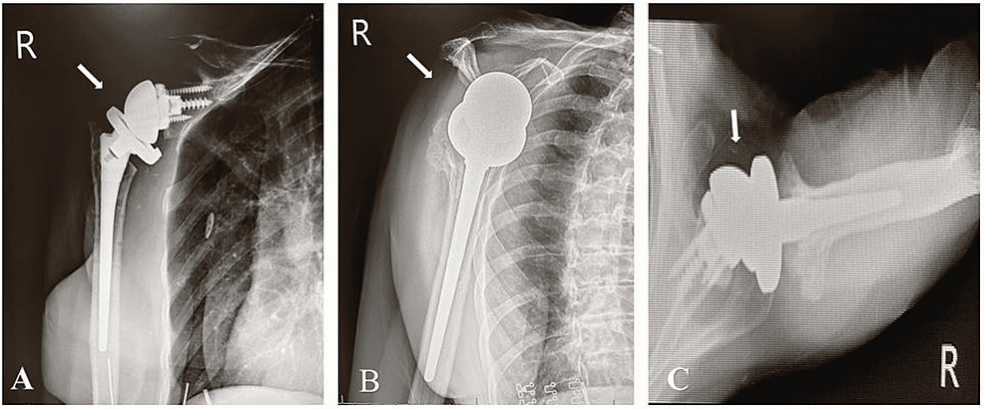
Three weeks post hardware removal and salvage reverse total shoulder arthroplasty X-rays of the right shoulder: (A) anteroposterior, (B) scapular Y, and (C) axillary views demonstrating intact and reduced glenohumeral joint prosthesis. No iatrogenic fractures visualized or no lucency around the prosthesis.

The patient underwent removal of hardware and rTSA 65 weeks after her previous hardware removal. Three weeks postoperatively, X-rays were remarkable for correct alignment of the right shoulder prosthesis (Figure 6). Twelve weeks postoperatively, the patient had improvement in her pain, active range of motion (AROM), and passive range of motion (PROM). (Table 1).

## Discussion

Multi-part PHL dislocations are challenging to treat. We present a case of a 64-year-old female who underwent primary ORIF for a three-part PHL dislocation. In this unusual presentation, the patient experienced multiple postoperative complications, requiring two additional operations over two years. All three operations were carried out by a single surgeon. There is a lack of cases reported in the current literature of late-onset AVN of the humeral head after hardware removal. Limitations of this case report include short-term follow-up from the final salvage rTSA surgery. There was also a lack of standardized patient reported outcomes to evaluate clinical outcomes postoperatively. 

The patient had no significant medical comorbidities that could affect fracture healing, like diabetes or osteoporosis [12]. The patient was relatively young and had a high pre-injury activity level. The primary goal of treatment was to maintain the patient’s pre-injury activity level and to avoid shoulder arthroplasty. Most existing studies evaluating the optimal treatment of acute PHF are retrospective [13]. The literature has demonstrated that ORIF compared to rTSA for displaced three-part or four-part PHF has lower complication rates and higher revision rates. ORIF and rTSA were shown to be comparable in their ranges of motion [14]. Shoulder arthroplasty should be avoided in younger populations to prevent the need for revision arthroplasty. Revision arthroplasty has higher complication rates and inferior functional outcomes compared to primary total shoulder arthroplasty [15]. rTSA compared to shoulder hemiarthroplasty for treatment for acute proximal humerus fractures has demonstrated superior long-term function scores and forward flexion. However, rTSA had a higher complication rate compared to hemiarthroplasty [16, 17]. Criteria intraoperatively that would have indicated the patient to have an rTSA over an ORIF was contingent on rotator cuff status and the amount of comminution that could affect screw fixation. Intraoperatively the patient was found to have an intact rotator cuff and proper reduction was achieved, allowing proper fracture fixation with ORIF. There was extensive comminution of the humeral head which contributed to the loss of humeral height. A fibular strut allograft was used to successfully achieve humeral height. Achieving an anatomic reduction and emergent treatment were factors that could have prevented the risk of AVN. The patient received two corticosteroid injections which could have contributed to her AVN, as corticosteroids have been shown to be a risk factor for AVN [18].

The patient had a complex fracture pattern. There is debate whether three-part or four-part fractures have a higher incidence of complications. A study by Trupka et al. found that dislocated and multi-fragmented PHF did not significantly increase the risk for AVN [19]. The dislocation associated with the fracture could have contributed to the compromise of the blood supply [19]. However, on initial presentation, the patient was vascularly intact on physical exam and the presentation of AVN occurred about two years after the initial injury.

The patient underwent salvage rTSA for improvement of her shoulder pain and range of motion. Outcomes of salvage rTSA compared to primary rTSA can be inferior [15]. A retrospective study completed by Ott et al. evaluated primary rTSA versus secondary rTSA after failed ORIF for PHF in elderly populations with an average age of 76 years. This study demonstrated statistically significant functional outcomes and range of motion for the primary rTSA group compared to the secondary rTSA group [20]. 

Salvage rTSA for failed primary ORIF has demonstrated satisfactory results. When counseling the patient for rTSA surgery, it was explained that it may improve their pain, but they may not regain their full shoulder range of motion. Factors contributing to this included degeneration of the rotator cuff from postoperative traumatic arthrosis, and arthrofibrosis of the shoulder capsule and surrounding soft tissues. A study done by Ernstbrunner et al. evaluated functional outcomes of patients younger than 60 years who underwent salvage rTSA from failed primary ORIF for proximal humerus fractures with a ten-year follow-up. This study demonstrated statistically significant improvement in mean subjective shoulder value scores, active forward flexion, abduction, pain scores, and strength after salvage rTSA [21]. In our case, the patient demonstrated improvement in her pain, AROM, and PROM 12 weeks postoperatively from salvage rTSA (Table 1).

## Conclusions

The treatment of complex PHF remains controversial. Further randomized control studies need to be completed to establish improved guidelines for the management of patients with complex PHF. These guidelines should take into consideration fracture patterns and patient characteristics.
